# Distinct Metabolic and Inflammation Signatures in Urban vs Rural Ugandan Youth With HIV on Dolutegravir

**DOI:** 10.1093/ofid/ofaf420

**Published:** 2025-07-18

**Authors:** Sahera Dirajlal-Fargo, Shan Sun, Kate Ailstock, Morgan Cummings, Nate Lucas, Rashida Nazzinda, Christine Karungi, Daisy Faith Oryem, Robert Kidega, Victor Musiime, Cissy Kityo, Grace A McComsey, Nicholas Funderburg

**Affiliations:** Department of Pediatrics, Northwestern University Feinberg School of Medicine, Chicago, Illinois, USA; Division of Pediatric Infectious Diseases, Ann and Robert Lurie Children's Hospital, Chicago, Illinois, USA; School of Health and Rehabilitation Sciences, Ohio State University School of Health and Rehabilitation Sciences, Columbus, Ohio, USA; School of Health and Rehabilitation Sciences, Ohio State University School of Health and Rehabilitation Sciences, Columbus, Ohio, USA; School of Health and Rehabilitation Sciences, Ohio State University School of Health and Rehabilitation Sciences, Columbus, Ohio, USA; Joint Clinical Research Centre, Kampala, Uganda; Joint Clinical Research Centre, Kampala, Uganda; Joint Clinical Research Centre, Kampala, Uganda; Joint Clinical Research Centre, Kampala, Uganda; Joint Clinical Research Centre, Kampala, Uganda; Department of Pediatrics, Makerere University, Kampala, Uganda; Joint Clinical Research Centre, Kampala, Uganda; Department of Pediatrics and Medicine, Case Western Reserve University, Cleveland, Ohio, USA; School of Health and Rehabilitation Sciences, The Ohio State University, Columbus, Ohio, USA

**Keywords:** dolutegravir, dyslipidemia, inflammation, insulin resistance, perinatally acquired HIV

## Abstract

**Background:**

In sub-Saharan Africa, the majority of the metabolic data are from youth living in urban areas. In youth with perinatally acquired HIV (YPHIV) and seronegative (HIV–), we examined inflammatory and metabolic signatures in urban versus rural Uganda.

**Methods:**

YPHIV (n = 100) were enrolled from urban and rural Uganda in an observational cohort study along with age- and sex- matched, population-based HIV– (n = 99) comparators. YPHIVs were on antiretroviral with HIV-1 RNA level ≤400 copies/mL. We compared variables using Wilcoxon rank-sum tests and chi-squared tests. General linear regression models were used to assess factors associated with metabolic and inflammatory biomarkers, adjusting for HIV status, socioeconomic factors, and other covariates.

**Results:**

Median age was 16.2 years, 52% rural versus 96% urban YPHIV had HIV-RNA <50 copies/mL, 93% of YPHIV were on Tenofovir, Lamivudine, and Dolutegravir. Overall, rural participants lived in extreme poverty compared to urban participants (*P* < .001). Urban YPHIV were more likely to have higher body mass index, Homeostatic Model Assessment for Insulin Resistance (HOMA-IR), total cholesterol, and low-density lipoprotein than rural YPHIV (*P* < .001); however sCD14, sCD163, high sensitivity C-reactive protein, interleukin-6, soluble tumor necrosis factor alspha receptor I (TNFRI), and lipopolysacchiride binding protein (LBP) were higher in rural YPHIV (*P* ≤ .001). After adjusting for demographic, socioeconomic, viral load and antiretroviral duration, only sCD14 remained elevated in the rural YPHIV (β: 1.1; 95% confidence interval, .2–2.0), and β D glucan in urban YPHIV (β 1.11; 95% confidence interval, .3–1.89).

**Conclusions:**

The monocyte activation marker sCD14, was associated with HIV status and remained elevated in rural YPHIV even after adjusting for differences in HIV factors. Increasing the inclusion of rural populations in sub-Saharan Africa is paramount as we focus on preventing comorbidities in aging YPHIV.

Pediatric HIV care in rural sub-Saharan Africa faces unique challenges, including delayed diagnosis and treatment initiation, and increased mortality rates compared to urban settings that may be due to limited healthcare infrastructure [1, 2].

Although much of the data on HIV-related chronic inflammation and downstream complications have been obtained from adults, the limited data that exist on immune activation in children or adolescents with perinatally acquired HIV (PHIV) in sub-Saharan Africa are primarily from urban areas and in children on older antiretroviral (ART) regimen [3–5]. In sub-Saharan Africa, it is estimated that 50%–60% of adolescents and young adults reside in rural areas [6]. In Uganda, that estimates is closer to 70% [7].

We have found that youth with and without PHIV in urban Uganda have ongoing systemic inflammation and immune activation [8], but disturbances in surrogate markers of intestinal integrity and microbial translocation persist only in youth with PHIV (YPHIV) despite viral suppression [9]. This supports the fact that ART does not fully restore the gut barrier even in younger populations.

The relationship between HIV, inflammation, metabolic risk factors, and differences in ethnicity, sanitation, diet, and physical activity in rural versus urban areas have not been thoroughly investigated in sub-Saharan Africa, home to 90% of YPHIV [10]. Extreme poverty is concentrated in Northern Uganda, which has experienced severe civil insecurity and resulted in mass displacement of people. There are also ethnic and dietary differences within Uganda [11, 12]. To further investigate these differences, we measured fasting lipids, insulin resistance (Homestatic Model Assessment for Insulin Resistance [HOMA-IR]), and plasma inflammatory and gut biomarkers in youth (gut integrity markers included the intestinal fatty acid binding protein, the fungal translocation marker β D glucan [BDG], and the microbial translocation marker lipopolysaccharide binding protein) with and without PHIV on a contemporary ART regimen from 2 separate sites in Uganda, a rural and urban site. We hypothesize that there are likely regional and ethnic variation in metabolic and inflammatory signatures in youth in Uganda; however, HIV-associated alterations may persist despite environmental factors. These regional differences are important to explore as PHIV are aging into adulthood and may be faced with increased risk of cardiovascular disease [13].

## METHODS

### Study Design

This is a cross-sectional analysis of data from an observational cohort study of PHIV and HIV seronegative (HIV–) youth prospectively enrolled at 2 distinct Joint Clinical Research Center sites: in Kampala (urban) and in Gulu (rural) Uganda between 2021 and 2023. All participants were 12–25 years of age. PHIV participants were on ART for at least 2 years with a stable regimen for at least the last 6 months with HIV-1 RNA <400 copies/mL. HIV– participants were either HIV– family members of the PHIV or recruited from the community using community liaison volunteers from the Joint Clinical Research Center. All HIV– patients were tested for HIV at study visit and were HIV unexposed and uninfected per report. Evidence of self-reported or documented diarrhea or acute infection (malaria, tuberculosis, helminthiasis, pneumonia, meningitis) in the last 3 months, as well as moderate or severe malnutrition were excluded. Participants who were pregnancy or intended to become pregnant were excluded.

#### Patient Consent Statements

The study was approved by the Research Ethics Committee in Uganda, the Ugandan National Council of Science and Technology, as well as the institutional review board of the University Hospitals Cleveland Medical Center, Cleveland, Ohio. Participants or caregivers (for children <18 years of age) gave written informed consent.

### Study Evaluations

Blood was drawn after an 8-hour fast, processed, cryopreserved, and shipped without prior thaw to University Hospitals Cleveland Medical Center, Cleveland, Ohio.

### Socioeconomic Measures

The primary parent or caregiver were given a 12-item questionnaire that incorporated the World Health Organization (WHO) STEPS instrument [14], and the Demographic and Health Surveys Wealth Index from US Agency for International Development [15], which assesses a household's cumulative living standard including types of water access and sanitation facilities. The questionnaire measured exposures of interest including the head of household's level of education, primary work or activity, and sources of income for the household, as well as overall food insecurity, access to electricity, and the primary water source over the past 3 months. Monthly income was converted to US dollars and divided by 30. Living below extreme poverty line was defined as living with <$2.15 per day as set by the World Bank as the International Poverty Line [16]. A binary variable was created to capture food insecurity (defined as frequency of hunger) compared to no food insecurity (ie, being hungry seldom or never). The primary water source was also dichotomized to capture a clean water source (piped into dwelling, open tap or protected stream or well) compared to an unsanitary source (unprotected stream, a stream without a point source or a public borehole). Dietary diversity was adapted from a questionnaire and scored accordingly [17].

### Metabolic Measures

Insulin was measured at a Clinical Laboratory Improvement Amendments–certified laboratory in real time in Uganda and the HOMA-IR was calculated as described [18], and insulin resistance was defined as HOMA-IR > 2.5 in Tanner stage 1 patients or >4.0 in Tanner stage ≥2. Body mass index (BMI)-for-age *z* scores were determined using WHO 2007 reference values, which is a reconstruction of the 1977 National Center for Health Statistics/WHO reference [19]. Physical activity was assessed using an activity questionnaire adapted from the National Institute of Allergy and Infectious Diseases Adult AIDS Clinical Trials Group Physical Activity Assessment [20, 21]. Metabolic equivalents of task (METs) were calculated. Physical activity requiring 3–6 METs were categorized as moderate intensity, whereas activities that required more than 6 METs were categorized as vigorous [22].

### Inflammation, Soluble Immune Activation, and Gut Markers

We selected biomarkers based on our previous findings in youth with and without HIV in Uganda. BDG (Mybiosource Inc., CA), is a polysaccharide cell wall component of most fungal species. Intestinal fatty acid binding protein (I-FABP, R &D Systems, Minneapolis, Minnesota, USA) is considered a marker of enterocyte inflammation or damage and associated with metabolic complications of HIV [23, 24]. Soluble CD14 (sCD14, R &D Systems) is a marker of monocyte activation. Soluble CD163, sTNFRI, high sensitivity C-reactive protein, and interleukin-6 were measured by enzyme-linked immunosorbent assay (R &D Systems; ALPCO, Salem, New Hampshire, USA; and Mercodia, Uppsala, Sweden). The intra-assay variability ranged between 4% and 8% and inter-assay variability was less than 10% for all markers. All assays were performed at Dr. Funderburg's laboratory at Ohio State University, Columbus, OH. Laboratory personnel were blinded to group assignments.

### Statistical Analysis

All variables were compared using Wilcoxon rank-sum tests and chi-squared test or Fisher exact test, where applicable.

We fitted general linear models, adjusting for age, sex, access to electricity, food insecurity, clean water source, dietary diversity, physical activity, HIV status, and site.

Orthogonal partial least-squares discriminant analysis (OPLS-DA) was conducted to distinguish urban from rural populations based on metabolic measurements and inflammatory and gut markers for the full cohort and PHIV separately. Volcano plots illustrate differences in metabolic outcomes and inflammatory markers between urban and rural groups. The x-axis represents the log2-transformed fold change (FC) of medians, whereas the y-axis shows the log10-transformed false discovery rate (FDR)-adjusted *P* values. A horizontal reference line at FDR *P* = .05 distinguishes statistically significant values (above the line, FDR *P* < .05). Two vertical reference lines at FC = 0.8 and 1.25 indicate thresholds for biologically relevant changes. FC refers to the ratio of medians between 2 conditions. An FC of 1.25 indicates that the level in the test group is at least 1.25 times higher than in the control group, whereas an FC of 0.8 indicates that the level in the test group is at least 20% lower than in the control group [25].

## RESULTS

### Participant Characteristics

Overall, 199 participants were included and had measurements for metabolic and inflammatory markers (99 HIV– and 100 PHIV). Median (interquartile range) age was 16 years (14, 17) and 103 (52%) were females.

Rural participants (50 PHIV, 49 HIV–) overall were slightly younger than urban participants (50 PHIV, 50 HIV–), with a median age of 15 versus 16 years. Viral suppression was lower among rural participants (52% rural vs 96% urban YPHIV had HIV-RNA <50 copies/mL), 93% of YPHIV were on Tenofovir, Lamivudine, Dolutegravir (Table 1). Rural participants had more socioeconomic adversity, they were more likely to live in extreme poverty, lack access to clean water, and had lower dietary diversity compared to urban participants (*P* < .001). Differences between the urban and rural HIV– participants are highlighted in Supplementary Table 1.

**Table 1.** Participant Characteristics by Site and HIV Status

	All Urban Participants (n = 100)	All Rural Participants (n = 99)	*P* Values	Urban PHIV (n = 50)	Rural PHIV (n = 50)	*P* Values
Age (years)	16 (15–18)	16 (141–18)	.009	16 (16–18)	15 (14–18)	.008
Female sex (%)	48 (48%)	55 (56%)	.286	24 (48%)	32 (64%)	.107
HIV variables						
Viral load <50 copies/mL (%)	48 (96%)	26 (52%)	.250	48 (96%)	26 (52%)	.250
CD4 cell count (cells/µL)	1013 (688–1387)	650 (456–872)	.004	1013 (688–1387)	650 (456–872)	.004
CD4	35 (29–43)	21 (16–25)	.003	35 (29–43)	21 (16–25)	.003
ART duration (months)	158 (132–173)	108 (87–148)	.000	158 (132–173)	108 (87–148)	.000
TDF/3TC/DTG	48 (96%)	45 (90%)		48 (96%)	45 (90%)	
Socioeconomic variables						
Lack of access to clean water	7 (7%)	46 (46%)	.000	5 (10%)	25 (50%)	.000
Lack of electricity	9 (9%)	93 (94%)	.000	5 (10%)	48 (96%)	.000
Food insecurity	0 (0%)	5 (5%)	.024	0 (0%)	5 (10%)	.023
Living in extreme poverty (%)	21 (21%)	85 (86%)	.000	10 (20%)	45 (90%)	.000
Caregiver education			.000			
No school	2 (2%)	1 (1%)				
Primary school	37 (37%)	83 (84%)		22 (45%)	48 (96%)	.000
Secondary school	44 (44%)	15 (15%)		21 (43%)	2 (4%)	
University	16 (16%)			6 (12%)		
Cardiometabolic risk factors						
Family history of cardiometabolic disease	22 (22%)	5 (5%)	.000	10 (20%)	2 (4%)	.014
Dietary diversity	7.0 (6.0–8.0)	6.0 (4.0–7.0)	.000	7.0 (6.0–8.0)	5.0 (4.0–7.0)	.000
Physical activity (total activity minutes)	1680 (1035–2040)	660 (356–1200)	.000	1830 (1050–2100)	645.0 (340–1110)	.000
Metabolic parameters						
Waist to hip ratio	0.81 (0.78–0.84)	0.83 (0.80–0.87)	.000	0.80 (0.78–0.85)	0.84 (0.81–0.88)	.001
BMI (kg/m^2^)	19.5 (17.8–20.9)	17.9 (16.1–19.6)	.000	19.2 (17.7–21.0)	17.4 (15.9– 9.0)	.000
BMI-for-age Z score	−0.6 (−1.1–−0.0)	−1.1 (−1.8–−0.5)	.000	−0.6 (−1.1–−0.1)	−1.4 (−2.0–−0.7)	.000
Systolic blood pressure (mm Hg)	66 (60–73)	67 (58–73)	.388	66 (60–73)	67 (56–73)	.425
Diastolic blood pressure (mm Hg)	113 (105–121)	112 (101–122)	.253	110 (104–119)	114 (100–122)	.419
HOMA-IR	2.10 (1.13–3.70)	1.16 (0.78–2.38)	.000	2.68 (1.69–3.97)	1.10 (0.78–2.06)	.000
Total cholesterol (mg/dL)	169.9 (142.1–204.5)	119.8 (97.2–139.2)	.000	156.7 (133.4–190.1)	107.4 (93.9–127.8)	.000
LDL (mg/dL)	103.3 (82.8–130.8)	65.9 (52.4–85.5)	.000	95.1 (83.0–122.6)	61.5 (44.4–80.2)	.000
Triglycerides (mg/dL)	88.9 (69.8–107.8)	79.1 (59.3–105.4)	.023	88.2 (66.3–110.9)	81.7 (59.1–112.6)	.215
Non-HDL cholesterol (mg/dL)	120.4 (98.7–148.7)	86.3 (72.1–101.4)	.000	108.7 (94.9–139.3)	80.1 (69.5– 95.0)	.000
Inflammatory and gut markers						
hsCRP (ng/mL)	361.5 (222.4–692.9)	1749.5 (357.2–5740.6)	.000	335.5 (199.2–735.7)	3313.8 (808.1–8248.2)	.000
IL-6 (pg/mL)	0.9 (0.6–1.5)	1.3 (0.7–2.5)	.004	0.9 (0.6–1.7)	1.8 (0.9–3.0)	.002
sTNFR-I (pg/mL)	780.9 (672.2–896.7)	872.5 (732.2–1068.7)	.000	805.0 (670.6–892.2)	883.2 (723.5–1101.5)	.003
sCD14 (pg/mL)	1422.9 (1197.0–1639.1)	1827.7 (1545.4–2157.6)	.000	1463.9 (1197.0–1665.2)	2059.0 (1739.0–2253.8)	.000
sCD163 (pg/mL)	615.2 (477.2–759.0)	935.3 (654.4–1573.1)	.000	629.9 (499.0–950.6)	1039.5 (745.0–1589.3)	.000
I-FABP (pg/mL)	2441.5 (1645.1–3657.6)	2589.0 (1555.8–3643.5)	.430	3241.0 (2130.6–5248.8)	2922.4 (2015.5–4197.4)	.205
BDG (pg/mL)	1729.3 (1424.8–2004.8)	1131.6 (885.2–1496.9)	.000	1775.1 (1554.7–2004.8)	1166.8 (881.0–1431.7)	.000
LBP (ng/mL)	10745 (6914.8–14642)	14150 (9787.0–17903)	.0004	11050 (7005.2–15479)	15424 (10053–19664)	.015

Medians (interquartile range).

Abbreviations: ART, antiretroviral therapy; BDG, Beta D Glucan, ; BMI, body mass index; HDL, high-density lipoprotein; HOMA-IR, Homestatic Model Assessment for Insulin Resistance ; hsCRP, high sensitivity C-reactive protein; I-FABP, intestinal fatty acid binding protein; IL-6, interleukin-6; LBP, lipopolysacchiride binding protein; LDL, low-density lipoprotein; PHIV, perinatally acquired HIV; TDF/3TC/DTG, Tenofovir, Lamivudine, Dolutegravir.

### Differences Between Urban vs Rural Sites

**Figure 1.**
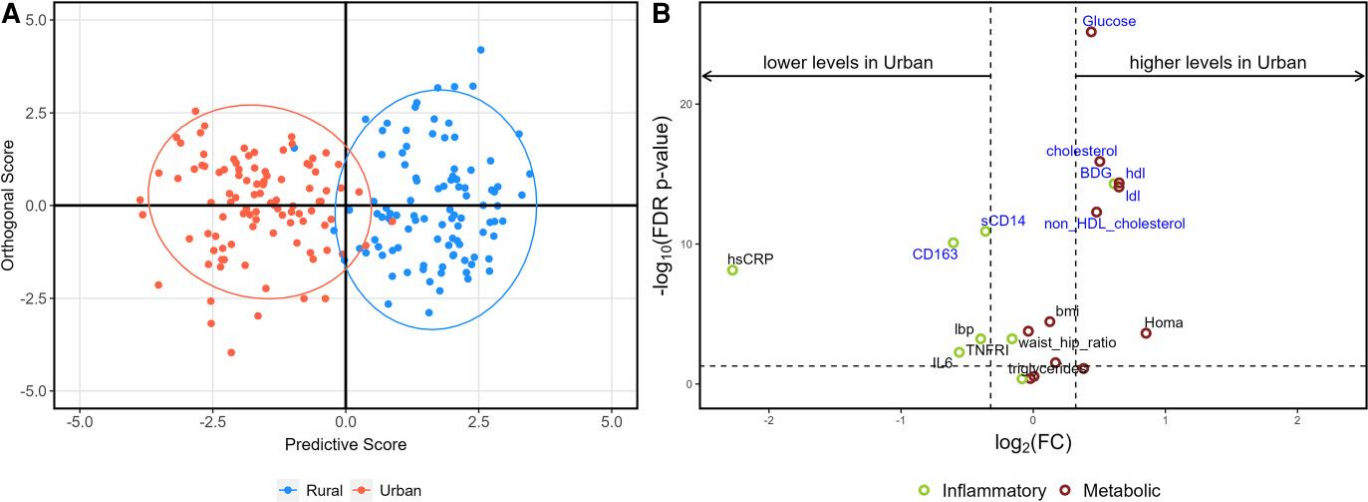
Orthogonal partial least squares discriminant analysis and volcano plots for all participants by sites. *A*, Orthogonal partial least squares discriminant analysis (OPLS-DA) demonstrating group discrimination based on metabolic outcomes and gut inflammatory markers between urban (red or the circle to the left) and rural (blue or the circle on the right) participants. *B*, Volcano plot illustrating the comparison of metabolic outcomes and inflammatory markers between urban and rural participants. The y-axis displays the log10 of the false discovery rate (FDR)-adjusted *P* values, whereas the x-axis represents the log2-transformed fold change (FC). The horizontal reference line (------) denotes the threshold for statistical significance, with points above the line being significant and points below considered not significant. The 2 vertical reference lines correspond to FC values of 0.8 and 1.25, respectively. All markers contributing to the differentiation between sites are labeled in blue.

OPLS-DA and volcano plot show variables that could discriminate between urban versus rural sites (Figure 1). This figure illustrates that rural and urban participants could be distinguished based on metabolic outcomes and inflammatory markers alone.

#### Metabolic Outcomes

Table 1 highlights that overall, urban participants had higher BMI, lower waist to hip ratio, and higher HOMA-IR and dyslipidemia compared to rural participants (*P* < .01). In Table 2, in adjusted regression analyses, non–high-density lipoprotein cholesterol remained significantly elevated in urban participants (β 0.85; 95% confidence interval [CI], .3–1.39]).

**Table 2.** Adjusted Models for Inflammatory Biomarkers and Metabolic Outcome

Factor	Parameter (95% CI) Adjusted by Urban Site —Full Cohort— [1]	Parameter (95% CI) Adjusted by Urban Site —PHIV— [2]
Metabolic outcomes		
HOMA-IR	0.30 (−.31 to .90)	0.44 (−.40 to 1.27)
Body mass index (kg/m^2^)	0.40 (−.12 to .91)	0.72 (−.09 to 1.54)
Non-HDL cholesterol (mg/dL)	0.85 (.3–1.39)**	0.39 (−.38 to 1.16)
Waist-to-hip ratio	−0.37 (−.96 to .22)	−0.18 (−1.02 to .66)
Inflammatory markers		
BDG (pg/mL)	1.37 (.88–1.85)***	1.11 (.3–1.89)**
sCD163 (pg/mL)	−0.40 (−.94 to .13)	−0.63 (−1.61 to .35)
I-FABP (pg/mL)	0.44 (−.13 to 1.02)	0.50 (−.61 to 1.61)
IL-6 (pg/mL)	0.00 (−.63 to .63)	0.04 (−.56 to .63)
sTNFR-I (pg/mL)	−0.27 (−.86 to .32)	−0.40 (−1.35 to .55)
hsCRP (ng/mL)	−0.00 (−.59 to .58)	−0.49 (−1.60 to .62)
LBP (ng/mL)	−0.14 (−.73 to .46)	−0.25 (−1.38 to .88)
sCD14 (pg/mL)	−0.45 (−.96 to .06)	−1.10 (−2.0 to −.20)*

Model 1: adjusted for age, sex, access to electricity, food insecurity, clean water source, dietary diversity, physical activity, and HIV status.

Model 2: adjusted for age, sex, access to electricity, food insecurity, clean water source, dietary diversity, physical activity, viral load, and ART duration.

Abbreviations: ART, antiretroviral therapy; BDG, Beta D Glucan, ; BMI, body mass index; HDL, high-density lipoprotein; HOMA-IR, Homestatic Model Assessment for Insulin Resistance ; hsCRP, high sensitivity C-reactive protein; I-FABP, intestinal fatty acid binding protein; IL-6, interleukin-6; LBP, lipopolysacchiride binding protein; LDL, low-density lipoprotein; PHIV, perinatally acquired HIV; TDF/3TC/DTG, Tenofovir, Lamivudine, and Dolutegravir .

Bolded values have significant *P* values, *P < .05; **P < .01; ***P < .001.

#### Inflammatory Markers

Markers of systemic inflammation, and microbial translocation were elevated in rural compared to urban participants (*P* < .01). BDG, however, was higher in urban participants (*P* < .01, Table 1). In separate regression models, after adjusting for sociodemographic variables, BDG (β 1.37; 95% CI, .88–1.85) remained elevated in urban participants (Table 2).

### Characteristics Between Urban vs Rural YPHIV

**Figure 2.**
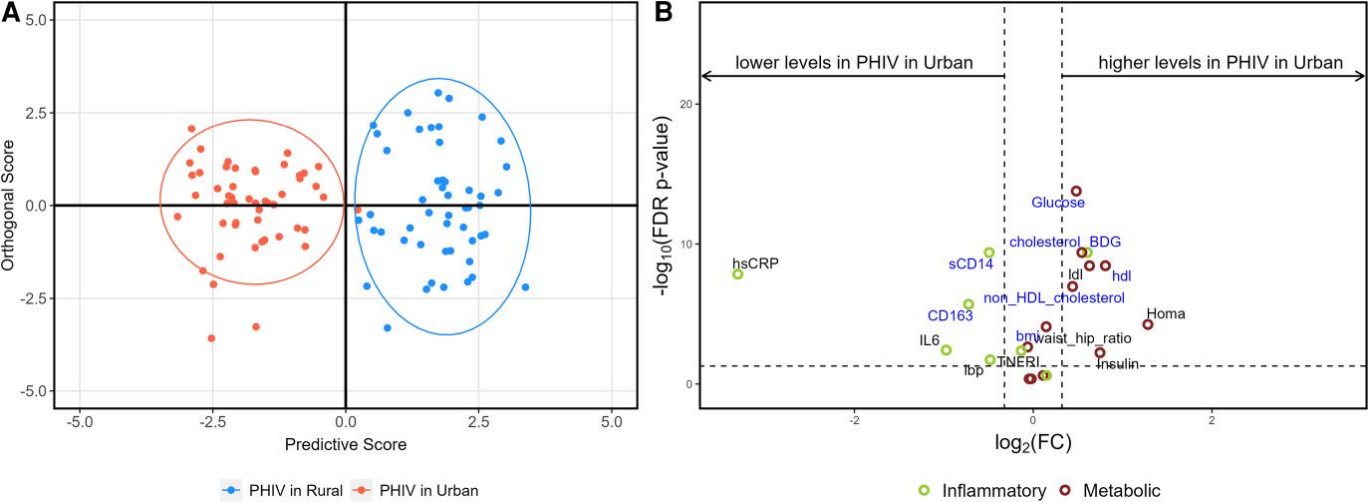
Orthogonal partial least squares discriminant analysis and volcano plots for perinatally acquired HIV (PHIV) participants only by site. *A*, Orthogonal partial least squares discriminant analysis (OPLS-DA) demonstrating group discrimination based on metabolic outcomes and gut inflammatory markers between urban (red) and rural (blue) PHIV participants. *B*, Volcano plot illustrating the comparison of metabolic outcomes and inflammatory markers between urban and rural PHIV participants. The y-axis displays the log10 of the false discovery rate (FDR)-adjusted *P* values, whereas the x-axis represents the log2-transformed fold change (FC). The horizontal reference line denotes the threshold for statistical significance, with points above the line being significant and points below considered not significant. The 2 vertical reference lines correspond to FC values of 0.8 and 1.25, respectively. All markers contributing to the differentiation between sites are labeled in blue.

OPLS-DA and volcano plot show variables that could discriminate between urban YPHIV versus rural YPHIV (Figure 2). This figure, compared to Figure 1, highlights that HIV appears to amplify the differences between rural and urban areas.

#### Metabolic Outcomes

Urban YPHIV were more likely to have higher BMI, HOMA-IR, total cholesterol, and low-density lipoprotein than rural YPHIV (*P* < .001, Table 1). Study site (urban vs rural) was no longer associated with any metabolic variables after adjusting for sociodemographic and HIV variables (Table 2).

#### Inflammatory Markers

Similarly to trends seen in all participants, biomarkers of systemic inflammation and microbial translocation, except for BDG, were higher in rural versus urban YPHIV (*P* ≤ .01, Table 1). After adjusting for demographic, socioeconomic, viral load, and ART duration, sCD14 remained elevated in the rural YPHIV (β –1.10; 95% CI, −2.0 to −.20) and BDG in urban participants (β 1.11; 95% CI, .3–1.89) (Table 2).

### Relationship Between Metabolic Outcomes and Inflammatory Biomarkers

We ran individual regression models, in urban versus rural sites, for each metabolic outcomes of interest, and investigated the associations for each inflammatory markers after adjusting for covariates as listed previously, including HIV status. We found that I-FABP remained associated with HOMA-IR in rural participants (β 0.0019; 95% CI, .0004–.0033)] and waist to hip ratio in urban participants (β 0.00001; 95% CI, .00000–.00001).

## DISCUSSION

In this study, of YPHIV and HIV seronegative youth in urban and rural Uganda, we found evidence of metabolic abnormalities in urban participants and elevated inflammation and gut integrity barrier dysfunction in rural participants, even after adjusting for demographic and socioeconomic differences. In analyses restricted to YPHIV participants, our findings were similar; however, after adjusting for HIV-specific variables such as HIV viremia and ART duration, only sCD14 remained elevated in rural YPHIV, and BDG in urban YPHIV. Our findings highlight that urban and rural participants have distinct socioeconomic, metabolic, and inflammatory signatures.

The poverty rate has fallen over time in Uganda, yet in 2020, according to the World Bank, 18% of Ugandans still live below international poverty line of $2.15 per day [26]. Additionally, despite improvements in the past decade, 23% lack access to drinking water, 17% lack sanitation, and 41% lack access to electricity [26]. Extreme poverty is concentrated in Northern Uganda, which has experienced severe civil insecurity and resulted in mass displacement of people. In accordance with this, we found that rural participants had more socioeconomic adversity: they were more likely to live in extreme poverty, lack access to clean water, and electricity, and parents/caregiver education was limited. These findings were consistent when limiting our analyses to YPHIV.

In Ugandan urban areas such as the capital, there is a nutritional transition, as seen in other urban African areas, with a shift to high consumption of rice and highly refined diets high in fat and sugar, but poor in fiber and micronutrient-rich foods [27, 28]. The Northern Ugandan diet, in rural areas, is mainly composed of plantain, starchy roots (cassava, sweet potatoes), and cereals (maize, millet, sorghum). Nuts and green leafy vegetables complement the diet. The intake of animal protein is typically very low [29]. Obtaining detailed data on household food access or individual dietary intake can be time consuming and expensive and requires a high level of technical skill difficult to achieve in rural settings. Dietary diversity is a qualitative measure of food consumption that allows to assess access to food variety and serves as a measure of nutrient adequacy [17]. We adapted a questionnaire from the Food and Agriculture organization to measure individual dietary diversity. We found that rural participants had lower dietary diversity both overall and in YPHIV only. We have previously found metabolic differences between HIV– and YPHIV in urban Uganda, specifically focused in and around the capital, Kampala, including decreased insulin sensitivity and dyslipidemia in YPHIV versus HIV– in Kampala [30, 31]. In this study, we also found that urban participants had evidence of metabolic complications; however, these differences were no longer significant when adjusting for sociodemographic, dietary, physical activity variables, and HIV variables when limiting our analyses to YPHIV only. These data should prompt additional attention to socioeconomic and dietary confounders when assessing cardiometabolic risk and the need for including rural populations especially in sub-Saharan Africa.

Although much of the data on HIV-related chronic inflammation has been obtained from adults, limited data exist on immune activation in children or adolescents with PHIV [32–35]. We have found that YPHIV in Uganda have ongoing systemic inflammation and immune activation [8], and associations with disturbances in surrogate markers of intestinal integrity and translocation in YPHIV despite viral suppression [9, 36]. This supports that ART does not fully restore the gut barrier even in younger populations. In this study, we find that rural participants have elevated markers of systemic inflammation, and microbial translocation. We found that sCD14, a soluble marker of monocyte activation that participates in the response of monocyte/macrophage cells to lipopolysaccharide, remains elevated in rural YPHIV even after adjusting for confounders. This is clinically significant as sCD14 is known to be associated with overall mortality and progression of atherosclerosis in HIV [37, 38]. Rural YPHIV participants in this study have a shorter duration of ART, lower CD4+ T-cell count, and more viremia, and regular HIV-RNA testing is often not feasible or accessible [39]. Soluble CD14 remained elevated in rural participants, despite adjusting for HIV (including higher rates of viremia in rural participants) and socioeconomic factors, and we hypothesize that the following factors may contribute to our findings: (1) differences in ethnicity: Bantu-speaking ethnic groups dominate the South and the North is home to the Nilotic and Central Sudanic-speaking groups; (2) likely differences in the microbiome: previous research in children in Europe versus a rural area in Burkina Faso, and in Malay children, suggest that children in rural Burkina Faso showed an enrichment in Bacteroidetes and depletion in Firmicutes and a greater abundance in Prevotella and Xylanibacter compared to European children, a profile that may be protective against inflammation and environmental enteropathy microbiota [40, 41]; (3) differences in exposures to parasitic infections, with schistosomiasis being endemic in rural Ugandan areas [42]; and (4) biomass cooking fuel exposure, which is more prevalent in rural settings [43].

In addition, we found that BDG, a marker of fungal translocation, was elevated in urban YPHIV participants. BDG is a component of the cell wall of many fungi, and we have previously found that BDG is elevated in urban Ugandan YPHIV compared to those who are HIV– [9, 44]. Adults living with HIV have altered mycobiomes [45–47]. Outside of HIV, altered mycobiome diversity has been associated with inflammatory bowel disease [48], atopic dermatitis [49], and chronic hepatitis B. Antifungal drugs improve gastrointestinal graft-versus-host disease [50]. Fungal translocation is not limited to the gut, and we cannot rule out an interaction between mycobiomes in different body sites such as the skin, oral, and nasal cavities, all of which are known to be colonized with fungi [51]. One hypothesis for evidence of elevated fungal translocation in urban PHIV is that fungal spores also account for large proportions of air particulate matter [52], and we have previously reported on high level of exposure to fine particulate matter in the urban cohort in Kampala [53].

Last, we found that the gut integrity marker I-FABP was associated with metabolic outcomes in both urban and rural settings. This supports the rest of our findings highlighting a role for alterations in gut integrity despite viral suppression on an integrase strand transferase inhibitor regimen. These findings are also consistent with our previous data both in US and Ugandan youth with PHIV [54], suggesting that gut permeability may contribute to metabolic derangements.

Strengths of our study includes age- and sex-matched participants with and without HIV from urban and rural settings in a high HIV prevalence setting with detailed socioeconomic and HIV history. Most PHIV participants were on contemporary ART with DTG. Therefore, our results can be generalized to rural and urban sub-Saharan African youth where most have been transitioned to a DTG-based regimen as first-line therapy. Limitations to our study include the lack of assessment of gastrointestinal microbiome and mycobiome composition. We are also limited by the cross-sectional nature of our analysis and small sample size for some of the groups. We also did not control for multiple comparisons.

In conclusion, our results from sub-Saharan Africa contribute to data highlighting the role of the environment, specifically in urban and rural settings, on metabolic and inflammatory signatures. These findings highlight the need to include rural populations in future studies, where the majority of youth reside in sub-Saharan Africa, especially because they are aging into adulthood and may face the long-term repercussions of sustained inflammation.

## Supplementary Material

ofaf420_Supplementary_Data

## Supplementary Data

Supplementary materials are available at *Open Forum Infectious Diseases* online. Consisting of data provided by the authors to benefit the reader, the posted materials are not copyedited and are the sole responsibility of the authors, so questions or comments should be addressed to the corresponding author.

## Notes

***Financial support.*** This work was supported by the National Institutes of Health (from the National Institute of Allergy and Infectious Diseases U01AI168630 to S.D.F. and N.F.; National Institute of Diabetes and Digestive and Kidney R21DK127522 to S.D.F.). This publication was made possible through funding support of University Hospitals Cleveland Medical Center Clinical Research Center (UHCRC) and the Clinical and Translational Science Collaborative of Cleveland, UL1TR004528 from the National Center for Advancing Translational Sciences (NCATS) component of the National Institutes of Health and NIH roadmap for Medical Research. Its contents are solely the responsibility of the authors and do not necessarily represent the official views of UHCRC or the NIH.

***Potential conflicts of interest.*** N.F. has received funding from Gilead. Grace McComsey served as a scientific consultant for Gilead, ViiV, and Merck, and has received grant support from Astellas, Tetraphase, Roche, Redhill, Pfizer, Cognivue, and Genentech. All other authors declare that the research was conducted in the absence of any commercial or financial relationships that could be construed as a potential conflict of interest.
