# Supplementary material for: Distinct Metabolic and Inflammation Signatures in Urban vs Rural Ugandan Youth With HIV on Dolutegravir
Source: Open Forum Infect Dis. 2025 Jul 18;12(8):ofaf420. doi: 10.1093/ofid/ofaf420 (PMC12341862; doi:10.1093/ofid/ofaf420)
Supplement: ofaf420_Supplementary_Data [file ofaf420_supplementary_data.docx]

|  | **Urban HIV- participants**  **(n=50)** | **Rural HIV- participants**  **(n=49)** | **P values** |
| --- | --- | --- | --- |
| Age (years) | 16.2 ( 15.1 - 17.9) | 16.0 ( 14.1 - 18.7) | 0.187 |
| Female sex (%) | 24 ( 48.0% ) | 23 ( 46.9% ) | 0.916 |
|  | **Socioeconomic variables** |  |  |
| Lack of access to clean water | 2 ( 4.0% ) | 21 ( 42.9% ) | 0.000 |
| Lack of electricity | 4 ( 8.0% ) | 45 ( 91.8% ) | 0.000 |
| Living in extreme poverty (%) | 11 ( 22.0% ) | 40 ( 81.6% ) | 0.000 |
| Caregiver education |  |  |  |
| no school | 2 ( 4.0% ) | 1 ( 2.0% ) | 0.000 |
| Primary school | 15 ( 30.0% ) | 35 ( 71.4% ) |  |
| Secondary school | 23 ( 46.0% ) | 13 ( 26.5% ) |  |
| university | 10 (20%) |  |  |
|  | **Cardiometabolic Risk Factors** |  |  |
| Family history of cardiometabolic disease | 12 ( 24.0% ) | 3 ( 6.1% ) | 0.013 |
| Dietary diversity | 8 (6 - 8) | 6.0 ( 5.0 - 7.0) | 0.000 |
| Physical Activity  (total activity minutes) | 1430 ( 990 -1920) | 660.0 ( 360.0 -1280.0) | 0.000 |
|  | **Metabolic Parameters** |  |  |
| Waist to hip ratio | 0.81 (0.78 - 0.84) |  |  |
| Body mass index (kg/m^2^) | 19.7 (17.9 - 20.9) | 18.9 ( 16.7 - 20.2) | 0.020 |
| BMI-for-age Z score | -0.5 (-1.1 - -0.0) | -0.9 ( -1.7 - -0.3) | 0.038 |
| Systolic blood pressure (mmHg) | 114.5 ( 107.0 - 123.0) | 111.0 ( 103.0 - 125.0) | 0.244 |
| Diastolic blood pressure (mm Hg) | 66.0 ( 61.0 - 73.0) | 66.0 ( 60.0 - 76.0) | 0.400 |
| HOMA-IR | 1.82 ( 1.01 - 3.23) | 1.55 ( 0.79 - 2.38) | 0.159 |
| Total cholesterol (mg/dL) | 185.4 ( 149.7 - 225.9) | 131.2 ( 108.7 - 146.4) | 0.000 |
| LDL (mg/dL) | 114.7 ( 82.6 - 139.1) | 77.5 ( 54.5 - 89.1) | 0.000 |
| Triglycerides (mg/dL) | 89.7 ( 71.5 - 104.1) | 75.3 ( 61.3 - 100.8) | 0.019 |
| Non-HDL cholesterol (mg/dL) | 130.2 ( 103.1 - 153.9) | 92.5 ( 77.5 - 106.8) | 0.000 |
|  | **Inflammatory and Gut Markers** |  |  |
| hsCRP (ng/mL) | 370.7 ( 234.7 - 596.6) | 804.1 ( 269.6 -2319.2) | 0.010 |
| IL6 (pg/mL) | 0.8 ( 0.6 - 1.4) | 0.8 ( 0.6 - 2.1) | 0.163 |
| sTNFR-I (pg/mL) | 777.1 ( 714.8 - 905.0) | 853.5 ( 740.5 -1010.4) | 0.021 |
| sCD14 (pg/mL) | 1370.4 (1198.2 -1618.3) | 1632.1 (1441.1 -1895.1) | 0.000 |
| sCD163 (pg/mL) | 596.0 ( 421.6 - 713.2) | 812.3 ( 584.7 -1379.2) | 0.000 |
| I-FABP (pg/mL) | 1837.6 (1506.6 -2658.9) | 1995.5 (1388.7 -2966.9) | 0.359 |
| BDG (pg/mL) | 1690.2 (1292.2 -1991.6) | 1092.5 ( 887.2 -1534.9) | 0.000 |
| LBP (ng/mL) | 10666 (6894.6 - 14348) | 13441 (9787.0 - 16766) | 0.005 |

**Supplemental Table 1**: HIV seronegative participant characteristics by site
